# Effect of Graphene Oxide Addition on the Properties of Electrochemically Synthesized Polyaniline–Graphene Oxide Films

**DOI:** 10.3390/polym16121677

**Published:** 2024-06-13

**Authors:** Armando Balboa-Palomino, Ulises Páramo-García, José Aarón Melo-Banda, José Ysmael Verde-Gómez, Nohra Violeta Gallardo-Rivas

**Affiliations:** 1 División de Estudios de Posgrado e Investigación, Tecnológico Nacional de México/Instituto Tecnológico de Ciudad Madero, Av. 1° de Mayo, Ciudad Madero C. P. 89440, Mexico; armando.bp@cdmadero.tecnm.mx (A.B.-P.); aaron.mb@cdmadero.tecnm.mx (J.A.M.-B.); nohra.gr@cdmadero.tecnm.mx (N.V.G.-R.); 2 División de Estudios de Posgrado e Investigación, Tecnológico Nacional de México/Instituto Tecnológico de Cancún, Av. Kabah km. 3, Cancún C. P. 77500, Mexico; jose.vg@cancun.tecnm.mx

**Keywords:** PANI, graphene oxide, cyclic voltammetry, composite, wettability, stability

## Abstract

In this work, the electrochemical synthesis of PANI and GO-modified PANI was performed using cyclic voltammetry, varying the amount of GO, 1 mg (PG1), 5 mg (PG5), and 10 mg (PG10) to analyze the effect of the amount of GO on the composite. PANI, PG1, PG5, and PG10 materials were characterized using optical microscopy, SEM, UV-vis, FTIR, Raman, and wettability. A stability test was also carried out by putting the materials to 500 oxidation-reduction cycles using cyclic voltammetry. The synthesis method allowed GO in PANI to be added through a chemical interaction between the two compounds. It was also found that the addition of GO led to an improvement in the hydrophilic character of the composite, which would lead to an improvement in the diffusion of reagents/species when the composites are used in aqueous media processes. The results of the stability test showed that the PG10 material presented a lower % loss of specific capacitance and energy compared with the other materials, which indicates that the GO presence (in the amount specified) improves the stability of the PANI. The PG10 material showed favorable and promising conditions for its use in fuel cell and battery processes.

## 1. Introduction

The discovery of conductive polymers in the 1970s gave rise to a wide range of possible applications due to the specific properties of these materials. Among many conductive polymers available today, polyaniline (PANI) is one of the most used and studied worldwide, mainly due to its good conductivity, good chemical stability, simplicity, and low production cost [1,2]. 

One of the most promising areas for the application of PANI is in the realm of g‘reen chemistry’, where it plays a crucial role in processes aimed at energy production and storage. For instance, it finds use in fuel cells [3], capacitors [4], supercapacitors [5], and sensors [6], among other applications, offering a sustainable solution to our energy needs.

While PANI boasts impressive properties, its standalone performance is not without its challenges, such as low mechanical stability and early degradation. This has spurred a wave of innovation in recent years, with the development of a diverse range of PANI-derived composites. These composites aim to enhance the material’s characteristics and properties, showcasing the dynamic and evolving nature of our field.

A composite combines two or more materials, generally of a similar nature, to obtain a product with better properties than the original materials separately. The choice of the material to be combined with the PANI will depend strictly on the properties to be improved, always bearing in mind the purpose or process in which the processed composite will be used.

The adaptability of PANI in composite materials is truly remarkable, with a wide array of combinations being explored. These include PANI with metal nanoparticles (PANI/NP’s) [7], mixed oxides (PANI/TiO2) [8], carbon-derived materials like carbon nanotubes (PANI/NTC) [9], graphene (PANI/G) [10], graphene oxide (PANI/GO) [11], reduced graphene oxide (PANI/rGO) [12], and many others. This extensive range of combinations is a evidence to the versatility and potential of PANI in various fields of materials science and electrochemistry.

GO, among the potential materials to form a composite with PANI, has sparked significant interest. This is because GO possesses key properties such as good electrical conductivity, mechanical stability, and hydrophilic characteristics due to the presence of oxygenated groups [13]. These properties, when combined with PANI, have the potential to overcome the limitations of PANI, leading to the development of materials with enhanced energy density and power, strength and durability, and improved hydrophilic character. This significant improvement in the overall results of these technologies has spurred further research to discover the best possible combinations for optimum performance in these materials.

PANI/GO materials have aroused great interest in electrochemical applications. In 2014, Hu et al. [14] performed the synthesis of a GO/PANI composite to evaluate the effect of GO on the electrochemical properties of the composite. The composite was prepared using chemical synthesis, and four different amounts of GO: 1.2%, 2%, 2.5%, and 3% were tested. The results obtained in the electrochemical tests indicated that the material with 2.5% GO presented a better specific capacitance value than the other materials (including pure PANI) and presented a higher stability by achieving 51% capacitance retention after 500 cycles, compared to 23% for PANI. These results were attributed to the good chemical synergy between the PANI and GO materials. 

In 2020, Gandara and Goncalves [15] electrochemically synthesized PANI composites with graphene oxide and reduced graphene oxide (rGO) as a coating for carbon fiber. This study focused on analyzing the method of composite synthesis. Four methods were performed as follows: (1) PANI electrosynthesis followed by GO dripping electrodeposition, (2) GO dripping followed by PANI electrosynthesis, (3) simultaneous electrodeposition using a mixture of PANI and GO, and (4) simultaneous electrodeposition using a mixture of PANI-rGO. The material synthesized using method (1) had the lowest impedance, resistivity, and the highest electroactivity. Condition (3) was the second best, with results slightly below condition (1). In this work, a quantity of 1 g of GO/rGO was used during the synthesis of the materials; this relatively high quantity caused inconveniences in conditions (3) and (4) because, in these conditions, the GO/rGO was in direct contact with the aniline in the mixture, which is considered to have caused a chemical oxidation of the aniline at the same time that the electrosynthesis was being carried out. This caused a decrease in the electrochemical formation of the polymer, which impacted the results of the techniques performed on the composites.

In this study, our primary focus is to explore the effects of different low amounts of GO added to PANI, aiming to observe the changes in properties due to the varying amounts of GO. We opted for a low amount of GO based on an extensive literature review and the personal experiences of our esteemed working group. A high quantity of GO has been found to cause interferences in the electrochemical synthesis of PANI, resulting in a composite with lower-quality properties than anticipated. Our indirect goal is to determine the optimal amount of GO to guide future research using PANI-GO. The PANI-GO thin films were synthesized using cyclic voltammetry in a conventional three-electrode cell. The materials obtained were meticulously characterized using a range of techniques including optical microscopy, scanning electronic microscopy, Fourier transform infrared spectroscopy, UV-visible spectroscopy, Raman spectroscopy, and wettability analysis. The electrical behavior of the obtained films was thoroughly analyzed through a stability test using cyclic voltammetry, paving the way for intriguing possibilities in future research.

## 2. Materials and Methods

### 2.1. GO Synthesis

The GO material was synthesized following to the methodology established by Guerrero y Caballero [16]. 1 g of synthetic graphite (<20 µm, 99.99% purity, Sigma Aldrich, St. Louis, MO, USA) and 0.5 g of sodium nitrate (NaNO_3_, Analytyka, Durham, NC, USA) were mixed in a flask. Subsequently, 23 mL of sulfuric acid (H_2_SO_4_, 98–99% purity) was added under constant stirring in an ice bath at 5 °C for 5 min; then, 3 g of potassium permanganate (KMnO_4_, Fermont, Fremont, OH, USA) was gradually added, and the mixture was maintained at 5 °C for 2 h. After that, the temperature was raised to 35 °C and kept there for 30 min under constant stirring. A total of 46 mL of deionized water was added, and due to the hydration heat, the temperature was raised to 98 °C. The bath was maintained at this temperature for 30 min under stirring. The reaction was finished by adding deionized water (140 mL) and hydrogen peroxide (10 mL at 10%). The resulting product was separated from the solution using centrifugation. The material obtained was washed using centrifugation at 6000 rpm for 20 min three times with 200 mL of dilute hydrochloric acid (HCl 5%) and hot deionized water (70 °C) to remove the remaining Mn and acid ions, respectively. Finally, the powders obtained were air-dried at 60 °C in an oven for 24 h.

### 2.2. PANI Synthesis

The obtention of PANI was carried out through cyclic voltammetry, using a 3-electrode conventional cell, a 1 cm of diameter and 3 cm of length acting as the working electrode (FTO plate, Sigma-Aldrich), stainless steel as the counter electrode, and Ag/AgCl as reference electrode. A mixture containing 0.3 mL of pure aniline (A.C.S., Fermont) and 19.7 mL of H_2_SO_4_ 0.5 M was used [17]. The mixture was submitted to 20 cycles, a sweep speed of 0.05 V/s, and a potential window from −0.2 V to 1.0 V. After the voltammetry, the obtained films were dried in an oven at 60 °C for 24 h. The average weight of the films obtained was 11 mg of PANI.

### 2.3. PANI–GO Synthesis 

The PANI–GO composite was synthesized under the same conditions as the PANI synthesis, except for an extra step, in which a dispersion of GO in deionized water was added to the cell containing the monomer and the electrolyte. Three different dispersions were made by adding 1, 5, and 10 mg of GO in 5 mL of deionized water. Hereafter, the materials obtained will be identified as PG1, PG5, and PG10. Performing the corresponding calculations based on the mass fraction of the mixture, it can be deduced that the PG1 material contains 0.32% wt of GO, the PG5 material contains 1.6% wt of GO, and the PG10 material contains 3.16% wt of GO. These percentages, although theoretical, are significant in understanding the material composition. The average weight of the PANI–GO films was 10 mg. The PANI and PANI–GO material syntheses were carried out using a Metrohm model AUTOLAB potentiostat/galvanostat.

### 2.4. Physical Characterization

In order to validate obtaining the GO, the material obtained was analyzed using Fourier transform infrared spectroscopy (FTIR) carried out in a Perkin Elmer^®^ Spectrum 100 spectrometer, with a built-in attenuated total reflectance module in a range from 4000 to 500 cm^−1^. The sample for this procedure was dried at 60 ° C for 72 h and measured by directly putting an amount of GO powder in the detector. We used X-ray diffraction (XRD) on a Bruker D8 Advance with a CuKα wavelength radiation λ = 0.15406 nm, equipped with a Lynx Eye detector. The sample was dried at 60 °C for 72 h before the test and was placed in a powder specimen holder for the test. Raman spectroscopy, using an i-Raman Plus equipment, BWTek brand, using a 532 nm laser radiation, with a nominal power of 35 mW, in a range of 60 to 4000 cm^−1^; the sample was dried at 60 °C for 72 h before the test and was measured directly without sample holder. Scanning Electron Microscopy (SEM) in a TESCAN equipment, model Vega 3SEM, using a voltage of 20 kV, the sample was dried at 60 °C for 72 h before the test; the GO powder dried was placed in an aluminum stub and coated with a small amount of Pd/Pt to improve signals.

PANI, PG1, PG5, and PG10 materials were analyzed using optical microscopy with a Maxlapter WR851 optical microscope, using a 4× lens and applying cold light above and below the sample, the film was directly measured from the FTO electrode. Scanning Electron Microscopy (SEM) in a TESCAN equipment, model Vega 3SEM, using a voltage of 20 kV, the sample was dried at 60 °C for 72 h before the test, the PANI-GO film in FTO substrate was coated with a small amount of Pd/Pt to improve signals. Ultraviolet spectroscopy (UV-vis) was performed on a G.B.C. Cintra 303, 300–800 nm range; PANI-GO films were dispersed in ethanol to conduct the measure. Fourier Transform Infrared Spectroscopy (FTIR) was carried out in a Perkin Elmer^®^ Spectrum 100 spectrometer, with a built-in attenuated total reflectance module ranging from 4000 to 500 cm^−1^, PANI-GO films on FTO were dried at 60 °C for 72 h and then directly measured. Raman spectroscopy, using an i-Raman Plus equipment, BWTek brand, using a 532 nm laser radiation, with a nominal power of 35 mW, in a range of 60 to 4000 cm^−1^, PANI-GO films on FTO were dried at 60 °C for 72 h and then directly measured. Finally, a wettability test was performed using the drop-based technique (using deionized water) in a THETA LITE 101 tensiometer. PANI-GO films on FTO were dried at 60 °C for 72 h and then directly measured.

### 2.5. Electrochemical Characterization

PANI, PG1, PG5, and PG10 films were tested through cyclic voltammetry to calculate the charge capacity of every material; for this purpose, H_2_SO_4_ 0.5 M was used as the electrolyte, a potential window from −0.2 V to 1.0 V and a scanning speed of 0.05 V/s. 

Subsequently, the materials were subjected to a stability/performance test, which consisted of 500 cycles of oxidation and reduction. Again, cyclic voltammetry and the same electrolyte and potential window were used, only the scanning speed was modified to 0.5 V/s. This analysis was carried out using a Metrohm model AUTOLAB potentiostat/galvanostat.

## 3. Results and Discussion

### 3.1. Graphene Oxide (GO)

**Figure 1:**
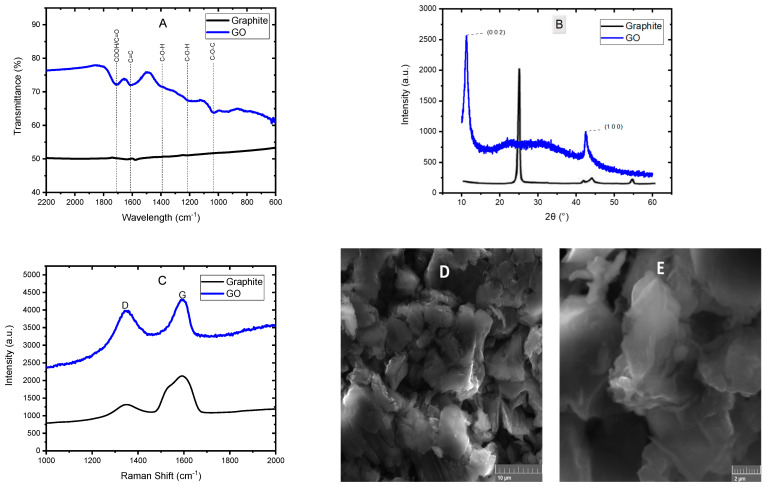
FTIR spectrum (**A**), XRD analysis (**B**), Raman spectrum (**C**) and SEM micrographs (**D**) and (**E**) of GO.

Figure 1 shows the results of the GO characterizations. The GO infrared spectrum is shown in Figure 1A, whereby it is possible to appreciate the appearance of signals attributed to the oxygen incorporation into the carbon structure, which indicates correct oxidation of the material during the synthesis process. The sign around 1040 cm^−1^ is attributed to the epoxy stretching mode (C-O-C) located on the basal plane of the graphene oxide. The bands at 1220 cm^−1^ and 1390 cm^−1^ correspond to the bending mode of the hydroxyl groups (C-OH) on the basal plane. The sign at 1620 cm^−1^ corresponds to C=C vibrations of the graphene skeleton. Finally, at 1710 cm^−1^, a signal corresponding to carbonyl functional groups (COOH/C=O) is observed at the edge of the graphene oxide sheets [16,18]. The graphite FTIR spectrum is also shown, the signals of the oxygen groups mentioned above are absent. In Figure 1B, the GO diffractogram is shown, in which two peaks can be seen, the first around 11° in the 2θ scale, which is characteristic of the crystalline plane (0 0 2) of the graphene oxide, while the second peak at 42° is typical of the crystalline plane (1 0 0) of the graphene oxide [19,20]. The usual peak of the graphite appears at 26°, so the fact that this peak does not appear in the diffractogram indicates the change in the material’s structure when oxidizing the graphite and obtaining graphene oxide. The Raman spectrum is shown in Figure 1C, in which two bands can be observed; the first, known as D-band (around 1340 cm^−1^), is associated with the imperfections in the graphene layers at the boundaries of the graphene particles and related to the alteration of aromatic hexagonal rings due to the existence of sp^3^ hybridizations including breaks in them. In contrast, the G-band (around 1590 cm^−1^), named the graphite band, and is associated with the stretching vibrations of the carbon atoms in the plane. In the graphite, the G-band is much more pronounced than the D-band, so this decrease in the intensity of the G-band indicates the good exfoliation of the graphite and the obtention of GO [21,22]. Lastly, the SEM micrographs of GO at 5000 and 15,000 magnifications are shown in Figure 1D and Figure 1E, respectively, in which the typical morphology of GO can be appreciated, which consists of a structure similar to wrinkled sheets or layers, thin and randomly aggregated, corroborating the oxidation of the multiple well-oriented layers of graphite [23,24]. These results confirm the good graphite oxidation and the obtention of GO.

### 3.2. Cyclic Voltammetry of PANI and PANI-GO

**Figure 2:**
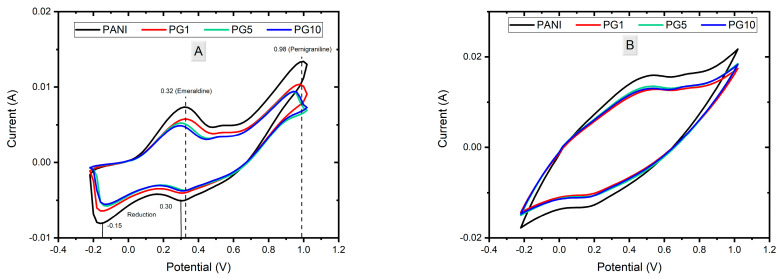
Voltammograms of PANI, PG1, PG5 and PG10 synthesis. Cycle 10 (**A**) and Cycle 20 (**B**).

The voltammograms of the synthesis of PANI, PG1, PG5, and PG10 materials are shown in Figure 2. Figure 2A shows cycle 10th of the synthesis of the materials in which the characteristic signs of polymer formation can be seen. The PANI material presents two peaks in the anodic part, the first around 0.32 V, corresponding to the aniline oxidation when changing from the leucoemeraldine form to the emeraldine form. The second peak, around 0.98 V, is attached to the oxidation of emeraldine to the pernigraniline form. In the cathodic part, two peaks are observed in 0.2 V and −0.15 V, which correspond to the reduction of PANI, as well as to the decrease in degradation products (p-benzoquinone/hydroquinone and p-aminophenol), respectively [25,26,27]. The PG1 material presents practically the same signals as the PANI material. Meanwhile, the PG5 and PG10 materials show a slight displacement to the left in the anodic signals, which can be attributed to the presence of GO in the system since, due to its good conductivity, the GO causes a better ionic transference in the medium, thus causing less potential to be needed to oxidize PANI. Likewise, the slight decrease in the signal or area under the curve of the GO-containing materials is attributed to the fact that GO competes with polyaniline for a place at the electrode/solution interface during synthesis, generating a modification in the particle diffusion in the mixture (in comparison to the PANI material), causing fewer aniline molecules to be available near the working electrode that can be polymerized [28]. 

Figure 2B shows the 20th cycle of the materials synthesis, in which the increase in the obtained sign and area under the curve can be seen. This indicates the correct growth of the polymer as the cycles increase. It can also be deduced that the PANI material will have a higher loading capacity value than GO-modified materials since the area under the curve is directly related to the specific capacitance of the material; the larger the area under the curve, the higher the capacitance. 

### 3.3. Optical Micrography and SEM

**Figure 3:**
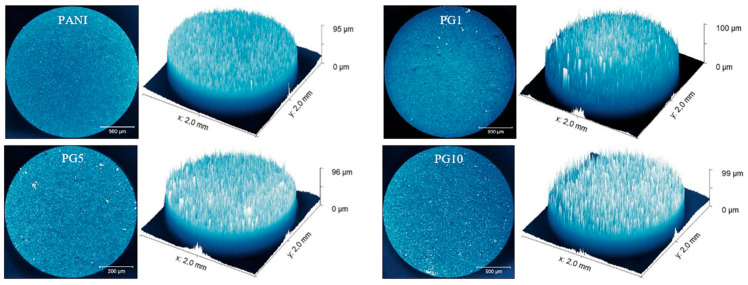
Micrographs and 3D profiles of synthesized materials.

The optical micrographs of synthesized materials are shown in Figure 3. A 3D cross-sectional profile accompanies these micrographs to visualize the film’s uniformity. The PANI material presents a better uniformity than the other three materials since the materials with GO show slight distortions in uniformity, which can be appreciated in the 3D cross-sectional analyses. This result is congruent with that obtained in the cyclic voltammetry analysis since the existence of GO in the solution during the film synthesis leads to a decrease in the concentration of available aniline near the surface of the working electrode, which leads to a reduction in the uniformity of polymer growth. Despite the mentioned, the variation in film uniformity of the GO-containing materials is very slight; no noticeable agglomerations are generated, and all films show very similar growth, so the film growth is satisfactory. 

**Figure 4:**
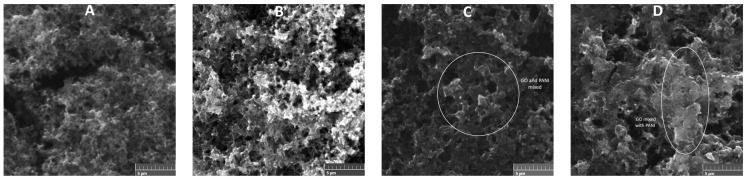
SEM micrographs of PANI (**A**), PG1 (**B**), PG5 (**C**), and PG10 (**D**).

The SEM micrographs of PANI, PG1, PG5, and PG10 are shown in Figure 4. The morphology of PANI (Figure 4A) displays a disordered and porous arrangement, typical of PANI, made up of granules and fibers, which proves that polymer growth occurs randomly [29]. PG1 material exhibits a morphology very similar to PANI without significant evidence of GO presence, which is attributed to the low GO load during the synthesis. On the other hand, the PG5 material (Figure 4C) begins to show a difference in morphology since indications of the presence of GO can be observed due to the appearance of a slight widening of the material’s fibers. This behavior is more noticeable in the PG10 material (Figure 4D), in which the mixture of both morphologies can be appreciated, and it can be taken as an indication of the combination and chemical interaction between both materials during the synthesis [30]. The materials with GO addition maintain a porous morphology, which is of great relevance since this type of structure facilitates the penetration of electrolyte ions and charges on the surface of the active material, improving electron transfer during the faradic reactions in which these materials commonly participate when used as electrodes either in capacitors or fuel cells [31].

### 3.4. UV-Visible, FTIR and Raman

**Figure 5:**
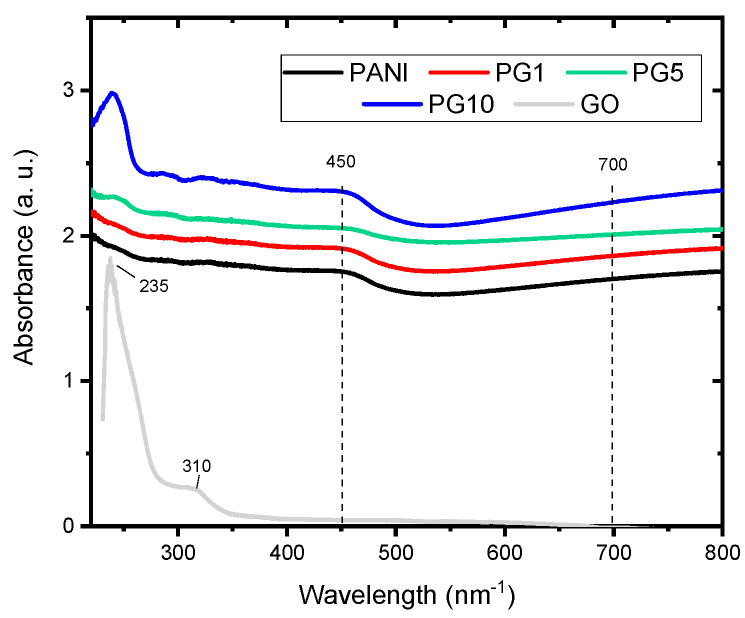
UV-vis spectra of synthesized materials.

The UV-visible spectra of the synthesized materials are shown in Figure 5. Two characteristic PANI signals are present in all PANI-GO materials, around 450 and 700 nm^−1^. The first band is attributed to the π–π* transition of a C-C aromatic bond, while the second absorption band is due to the formation of the polaron or bipolaron states for charged defects in the polyaniline [32,33]. The GO spectrum shows two typical signals around 235 and 310 nm^−1^, corresponding to π–π* transitions of C=C and n-π* transitions of C=O respectively [34]. The typical GO signal at 235 nm^−1^ does not appear in the PG1 material, which is attributed to the low GO loading, while in the PG5, material a small signal at that wavelength can be seen. In the PG10 material, the appearance of the signal is more noticeable (material with higher GO load). This is an indication of the formation of the PANI-GO composite.

**Figure 6:**
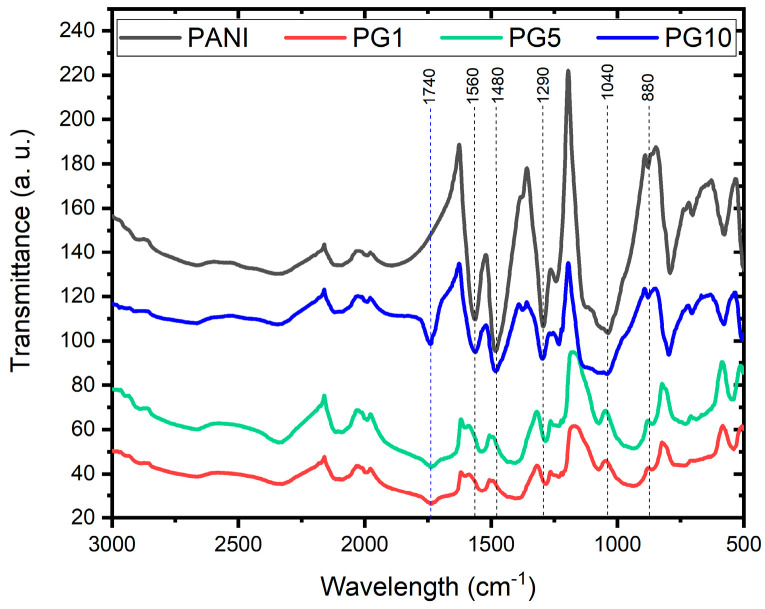
FTIR spectra of PANI, PG1, PG5 and PG10 materials.

Figure 6 shows that all the FTIR spectra have a similar profile, indicating the syntheses’ good. Analyzing the PANI spectrum, the signal obtained at 880 cm^−1^ corresponds to the vibration of the C-H bond outside the plane, which means a change in the angle of one of the characteristic C-H bonds of PANI. This signal also indicates branched polymer chains; in materials PG1 and PG5, the intensity of that signal decreased, indicating a slight perturbation in the structure of these materials due to the small amounts of GO used. The signal close to 1040 cm^−1^ identifies N-H^+^ bond stretching; meanwhile, at 1290 cm^−1^, a band representing the stretching of the C-N bond appears. The 1480 and 1560 cm^−1^ signals are characteristic of the quinoid ring’s C-C and C=N stretching vibration, respectively, revealing the PANI chains in their base emeraldine form [33,35,36]. The spectra of the GO-modified materials show small shifts and broadenings in the typical PANI signals, which is attributed to the presence of GO. The decrease in the intensity of the typical PANI signals in PANI-GO materials indicates the functional groups’ disappearance due to chemical reduction caused by the binding of PANI to GO. In addition to the mentioned above, the appearance of the signal around 1740 cm^−1^ in the PG1, PG5, and PG10 materials is indicative of the binding of the carboxyl group of GO to the nitrogen of the main chain of PANI [37]. Those mentioned above will indicate a chemical combination between PANI and GO during the synthesis, strengthening the argument exposed in the SEM analysis of the materials.

**Figure 7:**
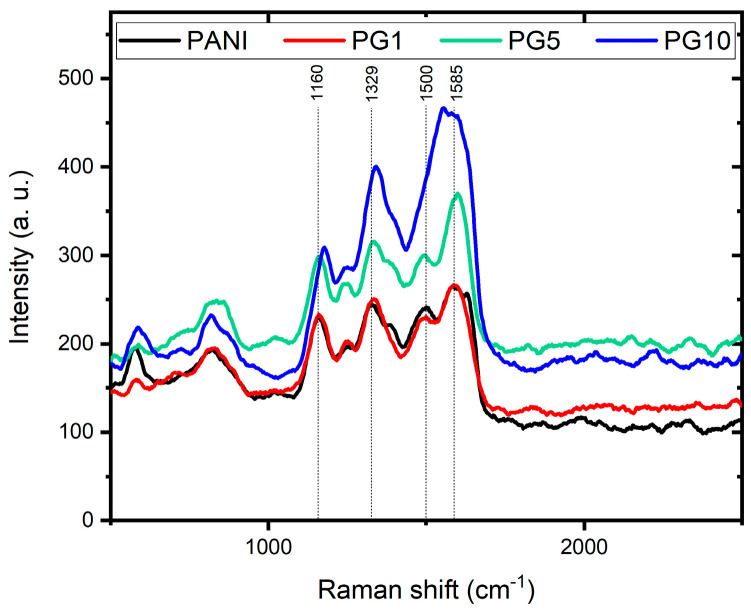
Raman spectra of PANI, PG1, PG5 and PG10 materials.

The Raman spectra of PANI, PG1, PG5, and PG10 materials are shown in Figure 7. In the spectrum of the PANI material, four signals can be appreciated, the first one at 1160 cm^−1^ corresponding to the C-H bending of the PANI quinoid ring, the second signal at 1329 cm^−1^ indicates the C-N^+^ stretching of the bipolaron, the third signal at 1500 cm^−1^ corresponds to the N-H bending of the bipolaron structure and the fourth signal at 1585 cm^−1^ indicates the C-C stretching of the benzenoid ring. These four signals corroborate the correct formation of PANI and agree with the literature [38].

The material’s spectra modified with GO show profiles similar to PANI’s, with a slight signal shift. Likewise, as the amount of GO in the materials increases, the typical signals attributed to PANI decrease, while the D and G signals typical of the GO (around 1340 and 1590 cm^−1^, respectively) appear in the spectrum. This behavior is evidence of the GO presence and its interaction with PANI, which is consistent with the results of FTIR. It is another indication of the formation of the PANI-GO composite [39]. 

### 3.5. Wettability

The results obtained from the wettability measurements of the processed materials are presented in Table 1. This analysis brings valuable information because it shows the affinity of a substrate with a liquid (water in this case), which is very important for the processes in which diffusion plays a key role. According to the literature, if the contact angle is between 10° and 90°, the material in question is considered hydrophilic, while a value above 90° would mean a hydrophobic material [40]. The results show that all the materials present a hydrophilic character. Moreover, the contact angle decreased as the amount of GO in the material increased. The decrease in the contact angle can be attributed to PANI being conductive polymer formed by alternating benzene rings and nitrogen atoms, which have a positive charge in their doped state. GO has different types of oxygenated species on its surface. These functional groups can interact, forming hydrogen bonds, electrostatic interactions, or even the potential to form covalent bonds with other molecules. Due to this, the PANI-GO composite becomes more hydrophilic than the pure PANI material. Table 1 shows that as the amount of GO increases, the decrease in the contact angle tends to a limit since, despite the functional groups mentioned above, there are also carbon chains, which naturally have a hydrophobic character.

This property of the synthesized materials is of great relevance since having a hydrophilic surface improves the transport of species from the electrolyte to the surface of the composite, which is one of the most critical points in the good performance of electrodes used for capacitors, battery, or fuel cell technologies [41,42].

### 3.6. Stability Test

**Figure 8:**
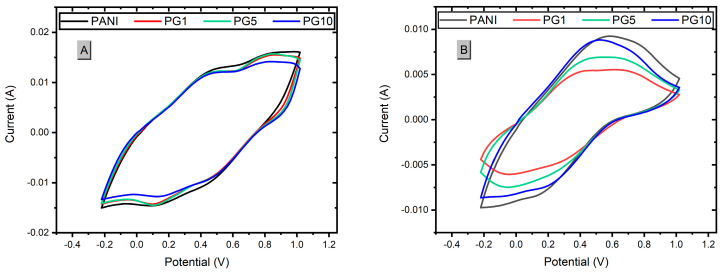
Voltammograms of materials before stability test (**A**) and after stability test (**B**).

The synthesized materials were subjected to a cyclic voltammetry test to obtain the necessary information to calculate the capacitance of the materials. After that, all the materials were proved in a stability test in which 500 cycles of charge-discharge (oxidation-reduction) were applied through cyclic voltammetry to simulate the attrition that the materials would have during their service life and to be able to calculate the loss of load capacity after such a test. Following this test, cyclic voltammograms were performed again on the materials to recalculate the capacitance of each one. Figure 8 shows these voltammograms.

Figure 8A shows the voltammogram of the materials, in which the area under the curve of the materials decreases slightly as the amount of GO added increases. There is a relationship between this area and the load capacity of the materials; the greater the area under the curve obtained, the better the load capacity. Considering the above as a reference, the PANI material presents the most significant area under the curve, making it the material with the highest load capacity (capacitance). The PG1 and PG5 materials present a similar area, although slightly smaller than the PANI. In contrast, the PG10 material presents the smallest area under the curve, indicating it has the lowest capacitance of the four materials. Due to these results, it can be concluded that the higher the amount of GO added to the synthesis, the lower the loading capacity of the PANI-GO film [40]. Figure 8B shows a decrease in the signal intensities and the area under the curve of the signals, as expected due to the wear to which they were subjected. It is also observed that PG1 and PG5 were the materials that lost more area than the area obtained before the stability test. At the same time, PG10 had a more minor loss than PG1 and PG5, which is an indication that the higher the amount of GO, the stability of the composite increases, which can be explained by the good mechanical properties of GO so that when the materials were subjected to wear, the material with more GO load withstood the wear better, thus causing the composite to have a lower loss of properties.

Based on the results obtained in the cyclic voltammetry, it is possible to calculate the specific capacitance (Cs) [F/g] using the following equation [11,31]:

Cs = ∫ idV/m∆VS(1)

where ∫ idV corresponds to the area under the curve, m is the electrode active mass [g], ∆V is the potential window used, V_final_ − V_initial_ [V], and S is the scanning speed [V/s]. Once the specific capacitance value is known, the specific energy (E) [Wh/kg] of the materials was calculated using the following equation:

E = 1/2(Cs)(∆V × 2)(2)

The results, crucial for our understanding, are presented in Table 2. It’s noteworthy that all the materials initially showed very similar Cs and E values. However, after the stability test, material PG10 demonstrated the lowest loss of Cs and E, followed by PANI, PG5, and PG1. This finding suggests that the addition of GO in PANI initially leads to a slight loss of stability compared to PANI. But as the amount of GO increased, the stability improved, reaching a point (10 mg of GO added) where the result was even slightly better than that of the PANI material. This significant result indicates that the addition of GO (10 mg GO) in PANI enhances the stability of the material compared to pure PANI, which is a major breakthrough considering the rapid degradation tendency of PANI. These results are in line with those reported in the literature for PANI and PANI-GO materials [43].

Comparing the specific energy results of the materials with the Ragone diagram, the PANI and PANI-GO materials would fall into the classification for use in batteries or fuel cells [11,43].

## 4. Conclusions

The addition of GO improved the hydrophilic character of the synthesized composites in comparison with the PANI material, being the most hydrophilic material and the one with the highest GO loading (PG10), which is a beneficial result since this implies a probable improvement in the diffusion of reagents in aqueous media, which is a vital point for possible applications of these materials as electrodes for capacitors, batteries or fuel cells.

Adding GO also improved the material’s stability, as the PG10 material obtained the lowest specific capacitance loss after being subjected to multiple oxidation-reduction cycles. This is very relevant since one of the biggest inconveniences of pure PANI is its lack of mechanical stability (rapid degradation) which limits its use as an electrode in some applications. This limitation could be avoided by adding a specific amount of GO to PANI.

It can be concluded that of the low amounts of GO added to PANI, the PG10 material (10 mg of GO) presented better results. In fact, it improved two key properties, such as wettability and stability, in comparison to the pure PANI material. This behavior could be further studied to achieve better materials for electrochemical applications.

The obtained values of the material’s specific capacitance and specific energy of the materials agreed with those reported in the literature. Compared with the Ragone diagram, they have a probable application as electrodes present in fuel cells or batteries.

## Figures and Tables

**Table 1:** Contact angles of PANI, PG1, PG5 and PG10 materials.

	PANI	PG1	PG5	PG10
Contact angle	36.4°	19.8°	18.9°	18.2°
Nature	Hydrophilic			

**Table 2:** Specific capacitance (Cs), specific energy (E), and % loss of the synthesized materials.

Material	Cs [F/g] Before Stability Test	(Cs) [F/g] After Stability Test	E [Wh/kg] Before Stability Test	E [Wh/kg] After Stability Test	% Loss
PANI	127.79	68.10	153.35	81.72	46.7
PG1	123.17	47.00	147.80	56.41	61.8
PG5	126.06	56.08	151.27	67.30	55.5
PG10	120.79	65.62	144.95	78.74	45.6

## Data Availability

The data presented in this study are available on request from the corresponding author.
